# *Glycine tomentella *Hayata inhibits IL-1β and IL-6 production, inhibits MMP-9 activity, and enhances RAW264.7 macrophage clearance of apoptotic cells

**DOI:** 10.1186/1423-0127-17-83

**Published:** 2010-11-05

**Authors:** Jia-Hau Yen, Deng-Jye Yang, Meng-Chi Chen, Yu-Fan Hsieh, Yu-Shu Sun, Gregory J Tsay

**Affiliations:** 1 Institute of Immunology, Chung Shan Medical University, Taichung, Taiwan; 2 School of Health Diet and Industry Management, Chung Shan Medical University, Taichung, Taiwan; 3 Department of Internal Medicine, Chung Shan Medical University Hospital, Taichung, Taiwan; 4 Department of Clinical Laboratory Chung Shan Medical University Hospital, Taichung, Taiwan

## Abstract

**Background:**

To assess the effects of *Glycine tomentella *Hayata (GTH), a traditional herbal medicine for treatment of rheumatic diseases on the expression of the proinflammatory cytokines and on the clearance of apoptotic cells by macrophages.

**Methods:**

RAW264.7 cells were cultured with lipopolysaccharide (LPS) in the presence or absence of ethanol extract of GTH. The expression of proinflammatory cytokines IL-1β, IL-6, and TNF-α, and inducible nitric oxide synthase (iNOS) and transglutaminase 2 (TG2) were assayed by reverse transcriptase-polymerase chain reaction (RT-PCR) and enzyme-linked immunosorbent assay (ELISA). Matrix metalloproteinase (MMP)-2 and MMP-9 were assayed by gelatin zymography. For detecting uptake of apoptotic cells, RAW264.7 cells were cultured with carboxyfluorescein diacetate (CFDA)-stained apoptotic cells and assayed by flow cytometry.

**Results:**

The major components of GTH analyzed by high-performance liquid chromatography (HPLC) chromatogram were daidzein (42.5%), epicatechin (28.8%), and naringin (9.4%).

GTH treatment inhibited the expression of proinflammatory cytokines IL-1β, IL-6 and MMP-9 but did not affect the expression of TNF-α and iNOS. GTH significantly enhanced the expression of TG2 and the clearance of apoptotic cells by RAW264.7 macrophages.

**Conclusions:**

GTH inhibits proinflammatory cytokine secretion and MMP-9 activity, enhances apoptotic cell uptake and up-regulates TG2 expression. Our data show that GTH might have beneficial effects on rheumatic diseases.

## Background

*Glycine tomentella *Hayata (GTH), also known as I-Tiao-Gung, is a plant of the soybean family. Root ethanol extracts of GTH have long been used as a traditional herbal medicine to treat a variety of rheumatic diseases, including rheumatoid arthritis (RA) and osteoarthritis (OA), in Kinmen, Taiwan [1-3]. Previous studies have documented an inhibitory effect of GTH on TNF-α expression by using a macrophage cell line of Atlantic salmon [2] and demonstrated analgesic and anti-inflammatory activities of the aqueous extract of GTH in mice [3]. GTH has also been reported to have anti-atherosclerotic effects and anti-oxidative activities [1,4]. However, the precise mechanism of the therapeutic effect of GTH on arthritis is not yet clear. It has not been investigated whether GTH affects the clearance of apoptotic cells or the production of matrix metalloproteinases (MMPs) and other proinflammatory cytokines.

RA is a common chronic inflammatory and destructive arthropathy characterized by the production of proinflammatory cytokines TNF-α, IL-1β, IL-6 and MMPs [5]. The etiology of RA remains enigmatic. Although biologic therapies for RA have dramatically changed over the past 20 years, some patients still fail to respond to treatments. The need for better therapies is as important as ever.

This study investigates the possible pharmacological functions and immunomodulatory effects of GTH. We found that GTH suppressed the expression of proinflammatory cytokines and MMP-9 activity, enhanced apoptotic cell uptake and up-regulated TG2 expression.

## Methods

### Ethanol extraction of *Glycine tomentella *Hayata (GTH)

GTH was a gift from the Kinmen Doctor Wang I-Tiao-Gung Company in Kinmen, Taiwan and authenticated by Professor Hsien-Cheh Chang of the China Medical University in Taiwan. The dry root of GTH (50 g) was grounded and extracted with 95% ethanol (500 ml) at a ratio of 1: 10 (wt/vol) and refluxed for 2 hours at 75°C twice. After evaporation of the organic solvent under reduced pressure, followed by lyophilization at 32.8°C, 3.7558 g of dry powder was obtained. The analytical equipments for the determination of flavonoids and phenolic acids in GTH by high performance liquid chromatography (HPLC) were a *PrimeLine™ *Gradient Model 500G HPLC pump system (Analytical Scientific Instruments, Inc., El Sobrante, CA, USA) with an injection valve (20 μL) (Rheodyne Inc., Cotati, CA, USA) and an S-3210 photodiode-array detector (PDA) (Schambeck SFD GmbH, Bad Honnef, Germany) [6].

### Cell culture of RAW264.7 cells

RAW264.7 cells were purchased from Bioresource Collection and Research Center (HsinChu, Taiwan) and cultured in Dulbeco's Modified Eagle Medium (DMEM) containing 10% fetal bovine serum (Biological Industries, Kibbutz Beit Haemek, Israel), 2 mM glutamine,1 mM pyruvate,1% non-essential amino acid,1000 U/ml penicillin, 0.0025 mg/ml amphotericin, and 1 mg/ml streptomycin (Biological Industries).

### Cell viability

Cell viability was assessed by the mitochondrial-dependent reduction of 3-(4,5-dimethylthiazol-2-yl)-2,5-diphenyl tetrazolium bromide (MTT) to purple formazan and cell death was determined with annexin V and propidium iodide staining. Cells were incubated with MTT (10%) for 4 hours and formazan crystals were dissolved in Dimethyl Sulfoxide (DMSO). The converted dye was quantified by absorbance at 570 nm. Measurement of early and late apoptosis was performed by flow cytometry using ANNEXIN V FITC KIT (AbD Serotec, Oxford, UK) according to the manufacture's instructions.

### Reverse transcription-polymerase chain reaction (RT-PCR)

Total RNA was isolated from RAW264.7 cells by using the Trizol reagent protocol (Sigma, St. Louis, MO, USA). Two micrograms of total RNA was denatured at 65°C with 1 μl oligo-dT (Promega, Madison, WI, USA), and 4 μl dNTPs (10 mM) for 5 minutes in 12 ml final volume. The primers-RNA mixture was cooled on ice, and 1 μl Moloney Murine Leukemia Virus (M-MLV) reverse transcriptase (Invitrogen, Carlsbad, CA, USA), 1 μl RNase inhibitor (Promega) and 4 μl 5× RT-buffer were added for a total volume of 20 ml. PCRs were performed under the following conditions: 94°C for 5 minutes, annealing at 54°C for 1minute, and DNA synthesis at 72°C for 2 minutes, followed by 28 cycles. To assess the effects of GTH on the mRNA expression of TG2 of RAW264.7 cells by RT-PCR, RAW264.7 cells were incubated with GTH (165 μg/ml) for 24 hours before RT-PCR. The amplified PCR products were subjected to electrophoresis in a 2% agarose gel.

Sequences for the PCR primers: 5'-TCCATGAGCTTTGTACAAGGA-3', 5'-AGCCCATACTTTAGGAAGACA-3' (forward and reverse mouse IL-1β probe), 5'-GTTCTCTGGGAAATCGTGGA-3', 5'-TGTACTCCAGGTAGCTA-3', (forward and reverse mouse IL-6 probe)5'-TGATGACCGGGAGGACATCA-3', 5'-GATTCTCCAGGTAGAGATCTC-3' (forward and reverse mouse TG2 probe), 5'-ATGAGCACAGAAAGCATGATC-3', 5'-TACAGGCTTGTCACTCGAATT-3' (forward and reverse mouse TNF-α probe), 5'-GCTCATGACATCGACCAGAA-3', 5'-ATCCACAACTCGCTCCAAGA-3' (forward and reverse mouse iNOS probe), 5'-TCACTCAAGATTGTCAGCAA-3', 5'-AGATCCACGACGGACACATT-3' (forward and reverse mouse GAPDH probe).

### Gelatin-zymography

To detect MMP-9 and MMP-2 activity, cell culture medium was collected and concentrated. Electrophoresis was performed using zymogram gelatin gels. Gelatin zymography was performed on a 10% sodium dodecyl sulfate-acrylamide gel containing 0.1% gelatin (SIGMA), rinsed in dd-H2O followed by incubation with bulky volume of renaturation buffer (2.7%TX-100 in dd-H2O) at room temperature for one hour with gentle shaking. The enzyme activity was developed in 50 mM Tris ph7.5, 0.2 M NaCl, 5 mM CaCl2 and 0.2% Brij35 at 37°C for 24 hours and stained with Coomassie Blue. MMP-9 and MMP-2 activity levels were normalized to that for β-actin. Images were obtained with an Alpha-Imager 2200.

### Phagocytosis assay

The human keratinocyte cell line (HaCat) was a gift from Professor Jen-Hung Yang of the Department of Dermatology at Chung Medical University in Taichung, Taiwan. For induction of UV-induced apoptosis, HaCat cells were exposed to UV radiation at 1650 J/m^2 ^by using the Spectroline UV Crosslinker with the Auto crosslink mode (Spectroline, New York, NY, USA). For phagocytosis of apoptotic cells, the UV-irradiated HaCat cells were labeled with carboxy-fluorescein diacetate (CFDA) followed by incubation with RAW264.7 cells at 10:1 target/macrophage ratio at 4°C for 4 hours. RAW264.7 cells were incubated with carboxylate-modified latex beads (Sigma, St. Louis, MO, USA) were used as the control. After washing, the cells on the dish were resuspended in Phosphate Buffer Saline (PBS) solution and 10,000-20,000 cells were analyzed for fluorescence intensity by flow cytometry. The phagocytosis index was calculated as the number of ingested beads or apoptotic cells divided by the total number of macrophages.

### Statistics

The data were analyzed with GraphPad Prism 4 software by one-way analysis of variance (one-way ANOVA) to determine the significance between sets of categorical data. A p-value of < 0.05 was considered to be significant.

## Results

### Contents of phytochemicals in GTH

**Figure 1:**
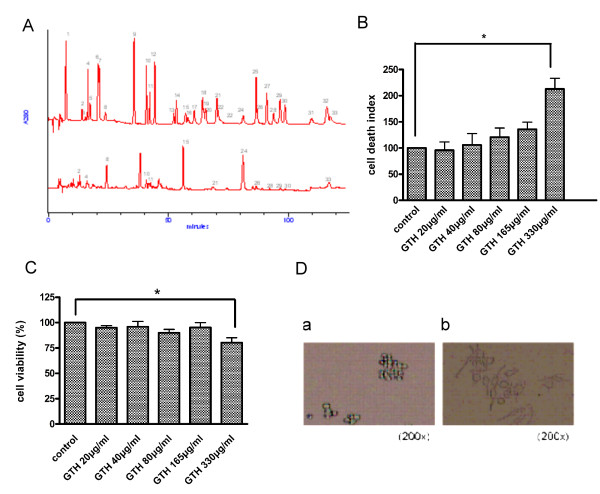
**HPLC separation and cell effects of GTH**. (A) HPLC separation of GTH. The established HPLC method with a C18 column was used for separation of the extracts of the roots of GTH. (B) Cytotoxic effects of GTH on RAW264.7 cells. RAW264.7 cells were incubated at different concentrations of GTH (20-330 μg/ml). The cell viability was measured by MTT tests (n = 3, *= P < 0.05). (C) Cell death with Annexin-V and PI staining analyzed by flow cytometry. Cell death was only induced at GTH concentration of 330 μg/ml. (D) Cell morphology after GTH treatment. RAW264.7 cells were treated with GTH at a concentration of 165 μg/ml for 24 hrs and observed by light microscopy. Photographs show the cells before (a) and after (b) GTH treatment.

The ethanol extract of GTH was analyzed by HPLC. Three phenolic acids including chlorogenic, ferulic and sinapic acids, and 11 flavonoids including catechin, epicatechin, naringin, riodictyol, daidzein, glycitein, narigenin, luteolin, genistein, hesperetin, and isorhamnetin could be determined in GTH. The major components of GTH were daidzein (42.5%), epicatechin (28.8%), and naringin (9.4%) (Figure 1A). The three components constituted 80.8% of the total GTH (Table 1 and Figure 1A).

**Table 1:** Content of flavonoids and phenolic acids in the ethanol extract of Glycine tomentella Hayata.3

**Peak no**.	Compound	retention time (min)	amount (mg/g extract)	**Peak no**.	compound	etention time (min)	amount (mg/g extract)
1	**Gallic acid**	7.59	ND	18	**Rosmarinic acid**	65.73	ND
2	**Catechin**	14.35	2.59 ± 0.10	19	**Quercitrin**	66.20	ND
3	**Gentisic acid**	15.65	ND	20	**Neohesperidin**	67.28	ND
4	**Chlorogenic acid**	16.81	0.60 ± 0.04	21	**Eriodictyol**	72.09	0.13 ± 0.01
5	**p-Hydroxy benzoic acid**	17.86	ND	22	**Diosmin**	72.54	ND
6	**Vanillic acid**	21.25	ND	23	**Morin**	73.69	ND
7	**Caffeic acid**	21.67	ND	24	**Daidzein**	83.23	12.02 ± 0.82
8	**Epicatechin**	24.54	8.16 ± 0.22	25	**Quercetin**	88.05	ND
9	**p-Cumeric acid**	36.63	ND	26	**Glycitein**	88.56	0.10 ± 0.01
10	**Ferulic acid**	41.64	0.46 ± 0.12	27	**Narigenin**	92.77	ND
11	**Sinapic acid**	43.04	0.42 ± 0.13	28	**Luteolin**	95.73	0.10 ± 0.01
12	**Syringic acid**	45.19	ND	29	**Genistein**	98.54	0.11 ± 0.01
13	**Rutin**	53.28	ND	30	**Hesperetin**	100.68	0.17 ± 0.01
14	**p-Anisic acid**	54.52	ND	31	**Kamempferol**	112.34	ND
15	**Naringin**	58.50	2.68 ± 0.09	32	**Apigenin**	118.91	ND
16	**Myricetin**	59.58	ND	33	**Isorhamnetin**	119.80	0.69 ± 0.02
17	**Hesperidin**	62.33	ND	Total			28.26

a. All values are mean ± SD obtained by triplicate analyses.

b. ND = not detected

### Cell viability

To assess the suitable concentration of GTH for the study, RAW264.7 cells were incubated with GTH at concentrations ranging from 20 to 330 μg/ml and cell viability was measured by MTT test 24 hours later. We found that at a concentration of 330 μg/ml, cell viability was reduced by 20% compared to the controls (Figure 1B; p < 0.05). The cell viability at GTH concentration of 20-165 μg/ml was no difference from that of the untreated controls. Figure 1C shows that cell death was induced at GTH concentration of 330 μg/ml by Annexin-V and PI staining. Therefore, the maximum concentration of 165 μg/ml was used for further experiments. After 24 hours of GTH treatment, the cells were observed under light microscope. The cell morphology had changed and their membranes had developed protrusions or processes which were in striking contrast to the round cells of the untreated controls (Figure 1D). The changes in cell morphology suggest activation of the cells by GTH stimulation.

### GTH regulates mRNA expression of proinflammatory cytokines

**Figure 2:**
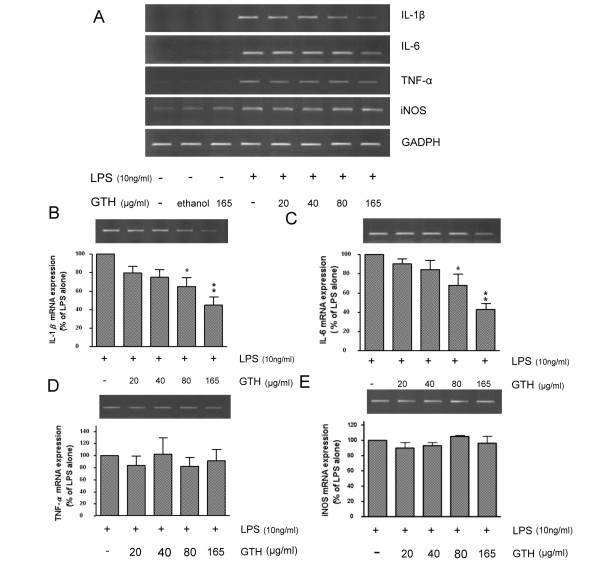
**GTH regulates cytokine mRNA expression**. RAW264.7 cells were incubated with GTH at 20, 40, 80, and 165 μg/ml for 24 hrs and stimulated with GTH LPS (10 ng/ml) for 4 hrs. (A) RT-PCR for mRNA expression of RAW264.7 cells treated with different concentrations of GTH, (B) IL-1β, (C) IL-6, (D) iNOS and (E) TNF-α mRNA at GTH 165 μg/m was quantified. Values represent mean ± SEM (n = 3, * = P < 0.05 ** = P < 0.001 compared with LPS alone)

To determine whether GTH affects the expression of proinflammatory cytokines, RAW264.7 cells were pre-treated with GTH at 20, 40, 80 and 165 μg/ml for 24 hours, followed by stimulation with LPS (10 ng/ml) for 4 hours. The mRNA expression was detected by RT-PCR (Figure 2A). GTH significantly reduced the expression of IL-1β and IL-6 by up to 55% and 56%, respectively (Figure 2B-C). However, GTH did not affect the mRNA expression of TNF-α and iNOS (Figure 2D-E). The inhibitory effect of GTH on IL-1β and IL-6 was exerted in a dose-dependent manner. In parallel, the experiments were also performed by using the human monocyte cell line U937 and we found that U937 cells pre-treated with GTH also decreased the expression of IL-6 mRNA in a dose-dependent manner (data not shown).

### GTH inhibits the production of IL-1β and IL-6

**Figure 3:**
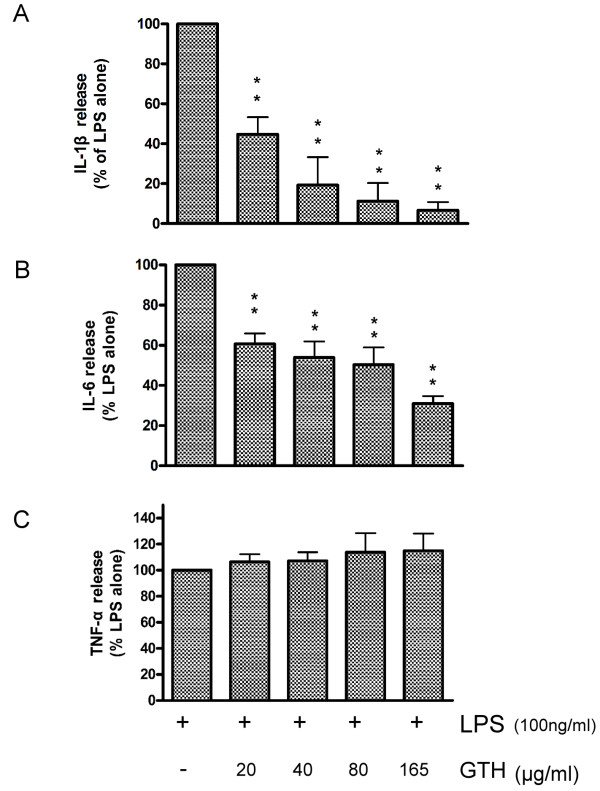
**GTH regulates cytokine protein production**. RAW264.7 cells were incubated and stimulated as described. Culture supernatant was analyzed by ELISA for (A) IL-1β, (B) IL-6, and (C) TNF-α. Values represent mean ± SEM (n = 3, ** = P < 0.001 compared with LPS alone).

RAW264.7 cells were incubated with GTH at 20, 40, 80 and 165 μg/ml for 24 hours before stimulation with 10 ng/ml LPS. The supernatants were collected 12 hours later and the cytokines were analyzed by ELISA. Consistent with the results that GTH treatment suppressed mRNA expression of proinflammatory cytokines by RT-PCR, the production of IL-1β and IL-6 were significantly decreased by GTH treatment (Figure 3A-B). In a dose-dependent manner, GTH at a concentration of 165 μg/ml inhibited IL-1β and IL-6 secretion by up to 93.2% and 79%, respectively. This data indicate that GTH is a potent inhibitor of IL-1β and IL-6. On the contrary, GTH did not reduce the expression and the production of TNF-α (Figure 3C).

### GTH down-regulates MMP expression

**Figure 4:**
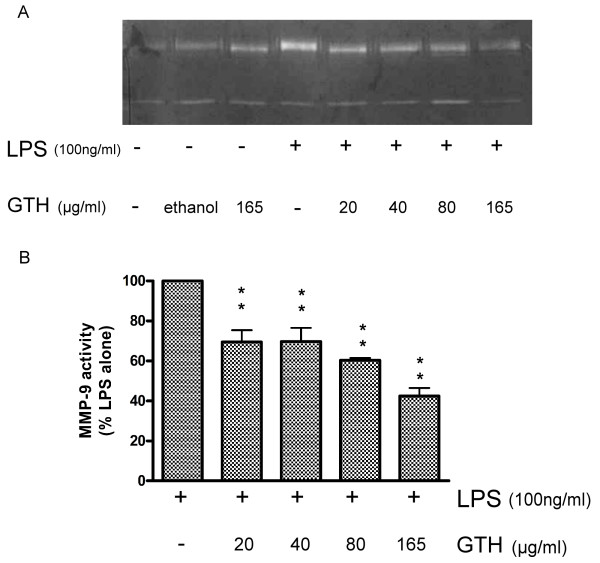
**GTH modulates the activity of matrix metalloproteinase**. RAW264.7 cells were incubated with different concentrations of GTH for 24 hrs, followed by stimulation with LPS. The activity of (A) MMP-9 and (B) MMP-2 was analyzed by gelatin zymography after 12-hr LPS stimulation. Values represent mean ± SEM (n = 3, ** = P < 0.001 compared with LPS alone).

To determine whether the ethanol extract of GTH affects the activity of MMPs in LPS-stimulated macrophages, RAW264.7 cells were incubated with different concentrations of GTH, followed by stimulation with LPS as described and the activity of MMP-2 and MMP-9 was analyzed by gelatin zymography. We found the activity of MMP-9, but not MMP-2, was significantly inhibited by GTH treatment in a dose-dependent manner (Figure 4A-B).

### GTH enhances clearance of apoptotic cells and the expression of TG2

**Figure 5:**
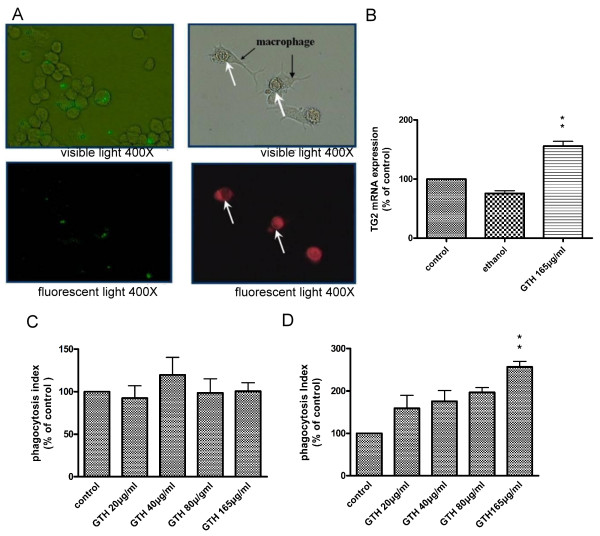
**GTH enhances phagocytosis of apoptotic cells**. RAW 264.7 cells were treated with GTH for 24 hrs and incubated with fluorescent latex beads or CFDA-labeled apoptotic cells. Photographs for (A) Uptake of latex-beads and (B) Phagocytosis of apoptotic cells were taken at 4 hrs. The upper panel shows the images by light microscope and the lower panel by fluorescent microscope. Black arrows indicate the macrophages and white arrows the apoptotic cells. In parallel, flow cytometry was used to detect the phagocytosis of (C) apoptotic cells and (D) latex beads. The data was expressed as phagocytosis index indicating the percentage of macrophages containing ingested beads or apoptotic cells. (E) TG2 mRNA expression after GTH treatment. Values represent mean ± SEM (n = 3, ** = P < 0.001 compared with control).

The findings that GTH down-regulated the expression and production of IL-1β, IL-6 and MMP-9 suggest that GTH may play a role in tuning down inflammatory responses. We further investigated whether GTH also plays a role in the clearance of apoptotic cells. Apoptotic cells were generated by UV irradiation of the keratinocyte cell line HaCat. The phagocytosis of the apoptotic cells by GTH-pretreated RAW264.7 cells was observed after a 4 hour co-culture by both light microscopy (Figure 5A) and by fluorescent microscopy (Figure 5B). In parallel, the phagocytic activity of macrophages was also detected with flow cytometry which showed that GTH strongly enhanced the macrophage clearance of apoptotic cells (Figure 5C). However the uptake of latex beads was not affected by GTH treatment (Figure 5D). To explore further the possible mechanism of the enhanced apoptotic cell clearance after GTH treatment, mRNA of GTH-treated RAW264.7 cells was analyzed for TG2 expression and a significantly increased TG2 mRNA expression was found in GTH-treated cells (Figure 5E).

## Discussion

Root extracts of GTH have long been used as a traditional herbal medicine to treat arthritis in Taiwan. GTH is also used as a functional tea, steeped wine and skin patch for commercial purposes because of its health advantages. In the present study, we demonstrated that the therapeutic effects of the ethanol extract of GTH on anti-proinflammatory cytokines are due to the suppression of IL-1β, IL-6 and MMP-9 activity. Previous studies documented that GTH has anti-inflammatory and analgesic activities [2,3]. The results of our study confirm and extend previous findings that GTH has immunomodulatory effects.

Recently, Chuang et al [2] reported that GTH could inhibit TNF-a in a macrophage cell line of Atlantic salmon but did not inhibit IL-1β. In contrast, we found that GTH could strongly inhibit IL-6 and IL-1β, but not TNF-a. The discrepancy of the effects of GTH may be due to the fact that the experiments used different cell lines and experimental systems. We pre-treated with GTH for 24 hours, then added LPS at a concentration of 10 ng/ml and 100 ng/ml for 2 hours and analyzed the cytokines by RT-PCR and ELISA. In contrast, Chuang et al had co-cultured GTH with LPS at a concentration of 2 mg/ml and analyzed the cytokines with real-time PCR. The discrepant effect of GTH on TNF-a need to be further clarified. Previous studies also demonstrated that daidzein decreased NO production in LPS-stimulated RAW264 cells [7] and inhibited iNOS expression and NO production in murine J774 cell line[8]. However, these studies were performed with individual and purified daidzein. According to the HPLC analysis, there are many different isoflavones in the extract of GTH. The effect of GTH may be different from that of the purified compounds. The discrepancies among these studies might also be caused by species differences.

It is interesting to note that the therapeutic range of GTH concentration for suppressing cytokines was relatively small. The viability of RAW264.7 cells was affected at GTH concentration of 330 mg/ml. While the effects of GTH on IL-1β, IL-6 and MMP-9 were seen at a lower concentration of 20 mg/ml, the effects of GTH on phagocytosis of apoptotic cells did not become apparent until the concentration of 165 mg/ml was used.

MMPs are involved in several pathological processes including cancers, inflammation and arthritis. Among the MMPs, MMP-9 has been shown to be involved in a variety of pathological processes of autoimmune diseases. MMP-9 secreted by macrophages regulates leukocyte migration in inflammatory diseases [9]. MMP-9 has also been shown to play an important role in cartilage degradation [9] and angiogenesis [10]. Our findings that GTH inhibit the activity of MMP-9 suggest the potential effect of GTH in mitigating the destruction of cartilage and inflammation of rheumatic diseases.

Recently, phagocytosis of apoptotic cells, also called efferocytosis, has attracted much attention because phagocyte clearance of apoptotic cells appears to be critical in the resolution of inflammation [11-13]. The ingestion of apoptotic cells by inflammatory macrophages also promotes the synthesis and release of anti-inflammatory mediators such as TGF-b1 and IL-10 [14-16]. By enhancing the clearance of apoptotic cells, GTH may be effective in resolving inflammation in arthritis.

Our results were identical to previous studies [4] that indicated that isoflavones, especially daidzein, were the effective components in *G. tomentella*. However, our study found that epicatechin and naringin were also the major compounds (flavonoids) in GTH in addition to daidzein. Moreover, there were few phenolic acids (chlorogenic, ferulic and sinapic acids) found in GTH.

## Conclusions

In conclusion, this study demonstrates that GTH enhances the clearance of apoptotic cells and is a potent IL-1β, IL-6 and MMP-9 inhibitor. These findings may explain the anti-inflammatory effects of GTH.

## Abbreviations Used

(GTH): *Glycine tomentella *Hayata;  (TG2): Transglutaminase 2;  (RA): Rheumatoid arthritis;  (OA): Osteoarthritis;  (MMPs): Metalloproteinases

## Competing interests

The authors declare that they have no competing interests.

## Authors' contributions

GJT initiated the concept and design of the study and collected, analyzed, and interpreted the data and prepared the manuscript. JHY, DJY, MCC, YFH, and YSS... collected the data. All authors read and approved the final manuscript.
